# Supplementary material for: Epstein–Barr Virus Infection of Oral Squamous Cells
Source: Microorganisms. 2020 Mar 16;8(3):419. doi: 10.3390/microorganisms8030419 (PMC7144007; doi:10.3390/microorganisms8030419)
Supplement: Supplementary file 1 [file microorganisms-08-00419-s001.pdf]

Supplementary figures

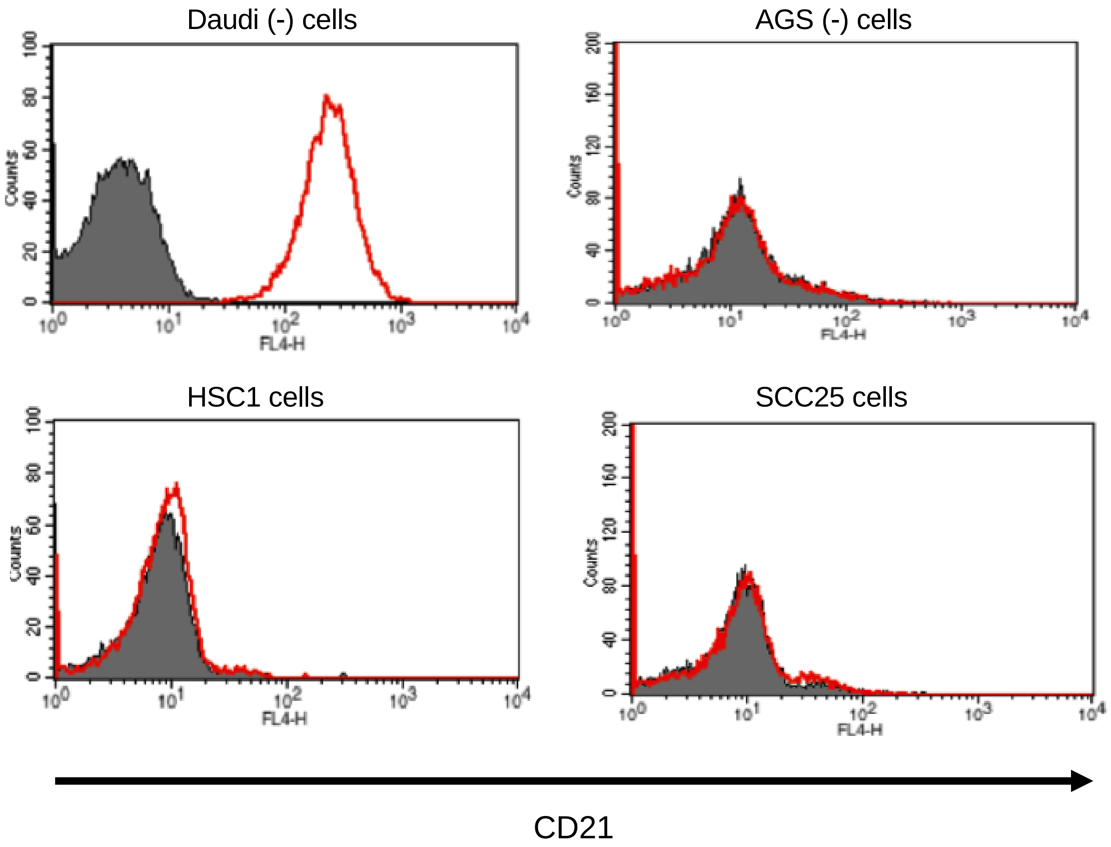

Figure S1. SCC cells do not express CD21. Cells were cultured overnight and collected, and CD21 expression was examined by flow cytometry. Daudi (-) cells were used as positive control and AGS (-) cells were used as negative control.

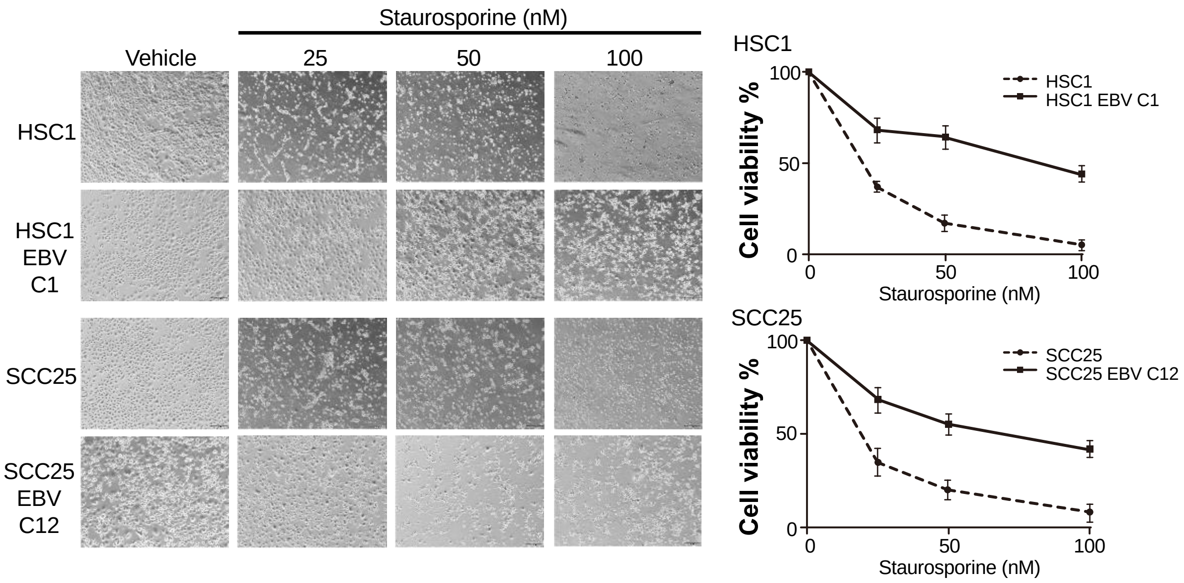

Figure S2. EBV infection attenuated apoptotic cell death of SCC cells. Cells were either treated or untreated with staurosporine at 0, 25, 50 and 100 nM for 24 h. Cell viability was examined by cell proliferation assay kit (WTS). Scale bar: 200 μm.

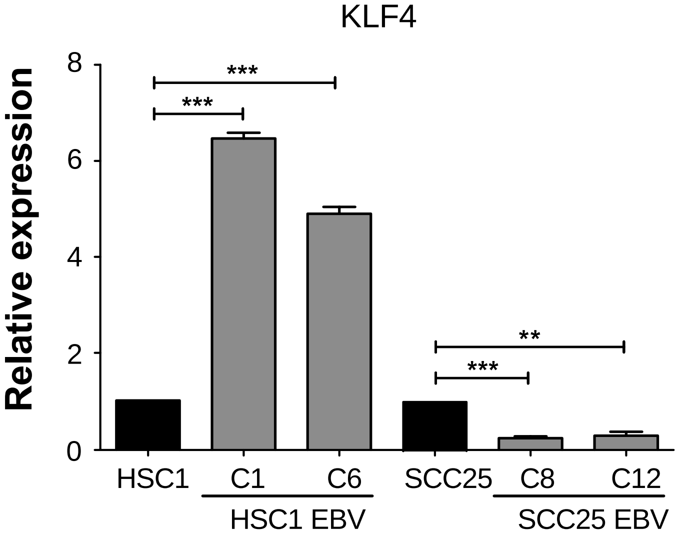

Figure S3. KLF4 is highly expressed in differentiated cells. Both EBV-positive and EBV-negative SCC cells were used to examine the expression of KLF4 by qRT-PCR.
